# Combinatorial approach for improved cyanidin 3-*O*-glucoside production in *Escherichia coli*

**DOI:** 10.1186/s12934-019-1056-6

**Published:** 2019-01-17

**Authors:** Biplav Shrestha, Ramesh Prasad Pandey, Sumangala Darsandhari, Prakash Parajuli, Jae Kyung Sohng

**Affiliations:** 1 Department of Life Science and Biochemical Engineering, SunMoon University, 70 Sunmoon-ro 221, Tangjeong-myeon, Asan-si, Chungnam 31460 Republic of Korea; 2 Department of BT-Convergent Pharmaceutical Engineering, SunMoon University, 70 Sunmoon-ro 221, Tangjeong-myeon, Asan-si, Chungnam 31460 Republic of Korea

**Keywords:** Multi-monocistronic, Anthocyanin, Cyanidin 3-*O*-glucoside, UDP-d-glucose

## Abstract

**Background:**

Multi-monocistronic and multi-variate vectors were designed, built, and tested for the improved production of cyanidin 3-*O*-glucoside (C3G) in *Escherichia coli* BL21 (DE3). The synthetic bio-parts were designed in such a way that multiple genes can be assembled using the bio-brick system, and expressed under different promoters in a single vector. The vectors harbor compatible cloning sites, so that the genes can be shuffled from one vector to another in a single step, and assembled into a single vector. The two required genes: anthocyanidin synthase (*Ph*ANS) from *Petunia hybrida*, and cyanidin 3-*O*-glucosyltransferase (*At*3GT) from *Arabidopsis thaliana*, were individually cloned under P_T7_, P_*trc*_, and P_*lac*UV5_ promoters. Both *Ph*ANS and *At*3GT were shuffled back and forth, so as to generate a combinatorial system for C3G production. The constructed systems were further coupled with the genes for UDP-d-glucose synthesis, all cloned in a multi-monocistronic fashion under P_T7_. Finally, the production of C3G was checked and confirmed using the modified M9 media, and analyzed through various chromatography and spectrometric analyses.

**Results:**

The engineered strains endowed with newly generated vectors and the genes for C3G biosynthesis and UDP-d-glucose synthesis were fed with 2 mM (+)-catechin and d-glucose for the production of cyanidin, and its subsequent conversion to C3G. One of the engineered strains harboring *At3GT* and *PhANS* under P_*trc*_ promoter and UDP-d-glucose biosynthesis genes under P_T7_ promoter led to the production of ~ 439 mg/L of C3G within 36 h of incubation, when the system was exogenously fed with 5% (w/v) d-glucose. This system did not require exogenous supplementation of UDP-d-glucose.

**Conclusion:**

A synthetic vector system using different promoters has been developed and used for the synthesis of C3G in *E. coli* BL21 (DE3) by directing the metabolic flux towards the UDP-d-glucose. This system has the potential of generating better strains for the synthesis of valuable natural products.
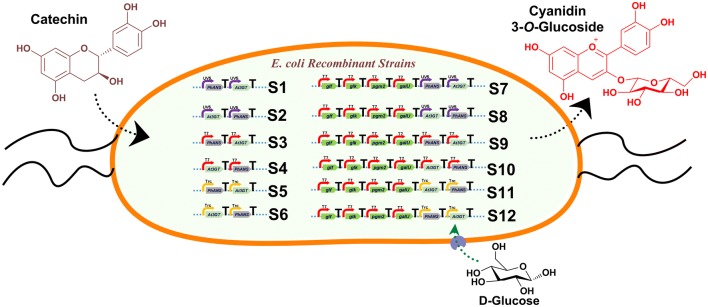

**Electronic supplementary material:**

The online version of this article (10.1186/s12934-019-1056-6) contains supplementary material, which is available to authorized users.

## Background

Phenolic compounds have potential antioxidant and anti-inflammatory properties, as well as other diverse health benefits when consumed as part of one’s diet [1–4]. Anthocyanins, the most important subclass of flavonoids and a major part of phenolic compounds, are highly colored plant pigments that are widely distributed in fruits and vegetables [5, 6], and are the products of the phenylpropanoid metabolism pathway. They are water-soluble glycosides of polyhydroxy and polymethoxy derivatives of 2-phenylbenzopyrylium or flavylium salts, which are responsible for the scarlet, magenta, purple, and blue colors of many fruits, vegetables, and cereal kernels [7], and also impart red colors to autumn leaves. In addition to the colors that they impart, anthocyanins, along with other flavonoids and phenolic acids, have attracted substantial attention, due to their beneficial health effects.

Anthocyanins are widely applied in the pharmaceutical industry and food industry as natural colorants, and are also used in cosmetic manufacturing. Recently, anthocyanins have been explored for their application as natural food colorants in beverages, dairy products, and snacks [8–10]. Some of the health benefits associated with anthocyanins include their protection against cardiovascular diseases; their anti-inflammatory, antioxidant, and anti-aging properties; and their role in the prevention of cancer [11, 12]. The health benefits of these plant metabolites have been attributed to not only their high antioxidant and antiradical activities, but also to other mechanisms, such as anti-mutagenesis, anti-carcinogenesis, and estrogenic activities; the inhibition of enzymes; and the induction of detoxification enzymes [13]. The antioxidant property of anthocyanins is associated with their ability to serve as free radical scavengers, which is attributed to their catechol ring backbones, which play important roles in their bioactivity [14].

The anthocyanin biosynthetic pathway has been extensively studied, and most genes encoding enzymes in the pathway have been isolated in many plant species. Anthocyanins are naturally synthesized from flavanones, such as naringenin or eriodictyol, starting with their conversion to dihydroflavonols by the enzyme flavanone 3β-hydroxylase (F3H) [15, 16], which are then converted by dihydroflavonol 4-reductase (DFR) through the reduction of the carbonyl group at the C4 position in order to form leucoanthocyanidins [17, 18]. The enzyme leucoanthocyanidin reductase (LAR) catalyzes the formation of a flavonol (+)-catechin [19], which is further converted into an intermediate compound cyanidin by the action of anthocyanidin synthase (ANS), a 2-oxoglutarate iron-dependent oxygenase [20]. ANS utilizes either catechins or leucoanthocyanidins as substrates to form cyanidin, an unstable intermediate, through a mechanism that comprises dehydrogenation, isomerization, and artificial dehydration in acidic conditions. Cyanidin eventually gets converted to anthocyanin through a C3 glucosylation reaction that is catalyzed by the activity of UDP-d-glucose flavonoid 3-*O*-glycosyltransferase (3GT) [21, 22], in order to produce cyanidin 3-*O*-glucosides (C3G) that are relatively stable under acidic conditions. In the recent past, extensive work has focused on different approaches toward the production of C3G biosynthesis in *Escherichia coli*. Various methods for increasing the C3G production titer have employed the optimization of pH of media, gene expression cassette, and the supply of UDP-d-glucose, along with induction optimization, and so on [23–25]. In recent years, there has been huge interest in the development of a system for increasing the production of C3G.

**Fig. 1:**
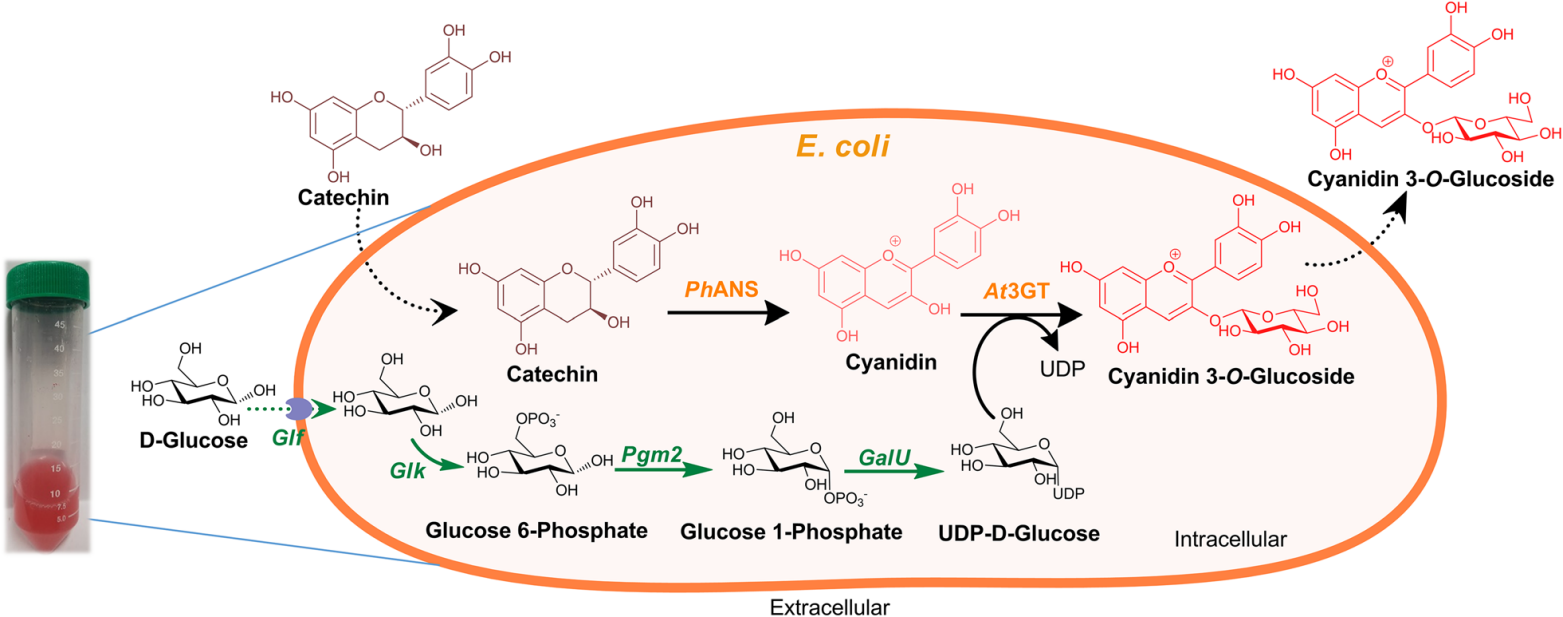
Engineered pathway for cyanidin-3-*O*-glucoside (C3G) biosynthesis from (+)-catechin using anthocyanidin synthase (*Ph*ANS) and 3-*O*-glycosyltransferase (*At*3GT) in *E. coli*. Also shown in the overexpression of the sugar biosynthesis genes

Various approaches, including the use of biological standard parts, such as BioBrick, have been extensively and routinely applied for the metabolic engineering of the pathways, and for the cloning of multiple genes. The *Bgl*Brick system uses the same principles of Biobrick with the use of isocaudomer pairs *Spe*I/*Xba*I, and *Bgl*II/*Bam*HI compatible cohesive ends, and is thus used for the expression of various proteins and genes with varying copy numbers and under different promoters of different strengths [26, 27]. In this study, we developed a multi-variate multi-monocistronic vector system based on a *Bgl*Bricks system, and used three different promoters P_T7_ (from previously constructed piBR181 vector [28]), P_*trc*_, and P_*lac*UV5_, in order to clone a total of six different genes, including the genes for UDP-d-glucose production and C3G production, in a combinatorial approach. For this purpose, an anthocyanidin synthase (*Ph*ANS: 1293 bp) gene from *Petunia hybrida* was selected because of its high catalytic activity, and flavonoid 3-*O*-glycosyltransferase from *Arabidopsis thaliana* (*At*3GT:1382 bp) was chosen for its regiospecific glucosylation of cyanidin. These two genes were synthesized, and cloned into these vectors, with four different genes (*glf*, *glk*, *pgm2*, and *galU*) for UDP-d-glucose synthesis in different combinations (Fig. 1), and the resulting amount of product formed was compared under identical conditions.

## Results

### Design and construction of expression vector piBRTrc and piBRUV5

Two multi-monocistronic operon system (piBRTrc and piBRUV5) were constructed for this study using the same strategy of *Bgl*Bricks, in which the bio-parts can be excised all at once, with a self-designing genetic circuit. A 382-bp ds-DNA construct containing the sequence of P_*trc*_ promoter, derived from the *E. coli lac*UV5 and *trp* promoter [29] and other bio-parts was computationally designed. The bio-parts consisted of five different restriction sites namely *Bgl*II/*Spe*I/*Hin*dII/*Bam*HI and *Xho*I including the *lac* Operator, Ribosome binding site (RBS) and rrnB T1 and T2 transcriptional terminators (Additional file 1: Figure S1) Similarly, a 172-bp ds-DNA construct containing sequence for P_*lac*UV5_ promoter which is a modified form of *lac* promoter [30] and other bio-parts was computationally designed. The bio-parts consisted of exactly the same restriction sites as that of piBRTrc vector, including the *lac* Operator, Ribosome binding site (RBS) and T7 transcriptional terminator (Additional file 1: Figure S2).

The sequences were synthesized and assembled in the *Bgl*II/*Xho*I site of the pET28a (+) vector using restriction digestion and the ligation of two different DNA fragments, as described in “Methods” section. The newly constructed *Bgl*Brick vectors take advantage of two isocaudomer pairs, *Xba*I/*Spe*I and *Bgl*II/*Bam*HI, which when digested generate a compatible cohesive end, and also form a scar sequence upon ligation that cannot be cleaved by their original restriction enzymes. The vectors have *Spe*I/*Hin*dIII cloning sites. However, the *Bgl*II restriction sites were present before the promoter, and *Bam*HI/*Xho*I were present after the transcription termination sites for multi-monocistronic operon assembly (Additional file 1: Figures S1, S2).

Using this vector system, it is applicable for multiple gene expression in multi-copy number transcriptome, so that a high yield production may be achieved. Because of the proximity of the promoter and the transcription termination sequences that are present at every end of the gene set, these vector systems may help in reducing the premature transcription termination and mRNA degradation. Moreover, the necessity of using multiple vectors for heterologous protein expression and use of various antibiotic creates stress to the culture, which can be reduced. However, the limitation is that the five restriction sites (*Bgl*II, *Spe*I, *Hin*dIII, *Bam*HI, and *Xho*I) must be absent from the gene sequence and the vector itself.

### Validation of newly generated vectors

In order to check the working efficacy of the newly constructed vector systems, we cloned two different genes encoding different proteins. The first gene was apramycin resistance gene (*apr*^r^), and the other was green fluorescence protein (*gfp*) encoding gene. Individual gene harboring plasmids were generated using a standard cloning protocol, and were checked for the expression of each gene. The expression of GFP protein was observed through visually monitoring the fluorescence under blue light. The expression of *apr*^r^ gene was checked by an antibiotic susceptibility test.

**Fig. 2:**
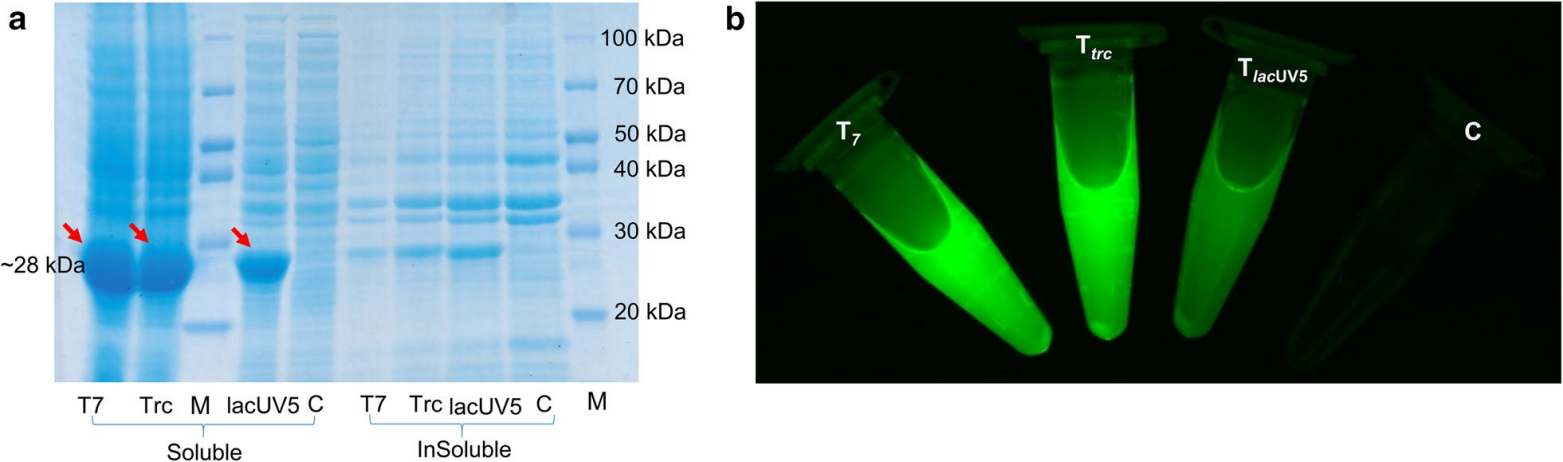
Functionality assay of newly constructed piBRTrc and piBRUV5 vectors (**a**) SDS-PAGE analysis showing the expression of *apr*^r^ in piBRTrc-apr^r^, piBRUV5-apr^r^, piBR181-apr^r^, and control (C) with empty pET28a (+) vector. **b** Visualization of *gfp* expression in piBR181-gfp, piBRTrc-gfp, piBRUV5-gfp harboring *E. coli* BL21 (DE3), and control (C) under blue light emission

The *E. coli* strains harboring the plasmids piBRTrc-Apr and piBRUV5-Apr (Additional file 1: Figure S3a) were constructed, and the expression of each gene was compared with *E. coli* BL21 (DE3) wild-type strain, *E. coli* BL21 (DE3) harboring pET28a (+) vector with kanamycin resistance gene, and *E. coli* BL21 (DE3) containing piBR181-Apr plasmid. Alternatively, the GFP protein was also cloned in the newly constructed vectors and pIBR181 to generate piBRUV-gfp, piBRTrc-gfp, and piBR181-gfp (Additional file 1: Figure S3b). All the strains with *apr*^*r*^ were subjected to disc diffusion assay with both kanamycin and apramycin. A clear zone of inhibition was observed in both antibiotics kanamycin and apramycin in the case of *E. coli* BL21 (DE3), whereas in the case of pET28a (+) vector, only a zone of inhibition in the presence of apramycin (up to 1 mg/mL) was observed, whereas no zone was visible in piBRTrc-Apr, piBRUV5-Apr, or piBR181-Apr, due to the presence of the apramycin resistance gene (Additional file 1: Figure S4). Further, the expression of *apr*^r^ gene in each of these three different plasmids by 12% SDS-PAGE analysis was accessed. The expression of the *apr*^r^ gene was observed at around 28 kDa (Fig. 2a). As expected, the highest amount of soluble protein was observed in piBR181-Apr, followed by piBRTrc-Apr and piBRUV5-Apr, showing the strength of each promoter. Additionally the GFP protein was expressed, and the fluorescence was checked under the blue light, in order to reconfirm the expression strength of the newly generated vectors with different promoters. A control strain of *E. coli* BL21 (DE3) harboring pET28a (+) was taken for reference. As anticipated, the intensity of GFP protein was the highest in piBR181-gfp containing *E. coli* strain, followed by piBRTrc-gfp and piBRUV5-gfp plasmid harboring strains (Fig. 2b). This result clearly shows the strength of each promoter in the expression of the protein, as expected. Moreover, the observed results further validated the two newly constructed plasmids as expression plasmids.

### Construction of C3G biosynthesis systems from catechin

An anthocyanidin synthase (*Ph*ANS: 1293 bp) gene from *Petunia hybrida* and flavonoid 3-*O*-glycosyltransferase from *Arabidopsis thaliana* (*At*3GT:1382 bp) was synthesized. Both genes were cloned in all three vectors having three different promoters; piBR181, piBRTrc, and piBRUV5 containing multi-monocistronic operon systems. Initially, *Ph*ANS and *At*3GT were cloned individually into piBR181, piBRTrc, and piBRUV5, and then assembled together in a multi-monocistronic fashion, in order to generate the desired recombinants, as described in Table 1. Meanwhile, the *Ph*ANS and *At*3GT genes were also shuffled back and forth to generate different recombinants for C3G production (Additional file 1: Figure S5). The cloning strategies for each gene are explained in the materials and methods.

**Table 1:** The different vectors, plasmids, and strains used in this study

Vectors and plasmids	Description	Source/references
pGEM^®^-T Easy	*E. coli* cloning vector, T7 and SP6 promoters, f1 ori, Amp^r^	Promega, USA
pET28 a(+)	*E. coli* expression vector, f1 pBR322 ori, Km^r^	Novagen, Germany
piBR181	Multi mono-cistronic vector modified from pET28a+, f1 pBR322 ori, Km^r^	Chaudhary et al. [28]
piBRTrc	Multi mono-cistronic vector modified from pET28a+, f1 pBR322 ori, Km^r^	This study
piBRUV5	Multi mono-cistronic vector modified from pET28a+, f1 pBR322 ori, Km^r^	This study
piBR181.Apr	piBR181 vector carrying apr^r^	This study
piBRTrc.Apr	piBRTrc vector carrying apr^r^	This study
piBRUV5.Apr	piBRUV5 vector carrying apr^r^	This study
piBR181.gfp	piBR181 vector carrying gfp	This study
piBRTrc.gfp	piBRTrc vector carrying gfp	This study
piBRUV5.gfp	piBRUV5 vector carrying gfp	This study
piBR181-*Ph*ANS	piBR181 vector carrying *Ph*ANS	This study
piBR181-*At*3GT	piBR181 vector carrying *At*3GT	This study
piBR181-*Ph*ANS.*At*3GT	piBR181 vector carrying *Ph*ANS.*At*3GT	This study
piBR181- *At*3GT. *Ph*ANS	piBR181 vector carrying *At*3GT.*Ph*ANS	This study
piBRTrc-*Ph*ANS	piBRTrc vector carrying *Ph*ANS	This study
piBRTrc-*At*3GT	piBRTrc vector carrying *At*3GT	This study
piBRTrc-*Ph*ANS.*At*3GT	piBRTrcvector carrying *Ph*ANS.*At*3GT	This study
piBRTrc- *At*3GT. *Ph*ANS	piBRTrc vector carrying *At*3GT.*Ph*ANS	This study
piBRUV5-*Ph*ANS	piBRUV5 vector carrying *Ph*ANS	This study
piBRUV5-*At*3GT	piBRUV5 vector carrying *At*3GT	This study
piBRUV5-*Ph*ANS.*At*3GT	piBRUV5 vector carrying *Ph*ANS.*At*3GT	This study
piBRUV5- *At*3GT. *Ph*ANS	piBRUV5 vector carrying *At*3GT.*Ph*ANS	This study
pIBR181-glf.glk.pgm2.galU	piBR181 vector carrying glf.glk.pgm2.galU	Parajuli et al. [31]
piBR181-glf.glk.pgm2.galU.*Ph*ANS.*At*3GT	piBR181 vector carrying glf.glk.pgm2.galU.*Ph*ANS.*At*3GT	This study
piBR181-glf.glk.pgm2.galU.*At*3GT.*Ph*ANS	piBR181 vector carrying glf.glk.pgm2.galU.*At*3GT.*Ph*ANS	This study
piBRTrc-glf.glk.pgm2.galU.*Ph*ANS.*At*3GT	piBRTrc vector carrying glf.glk.pgm2.galU under T7 promoter and *Ph*ANS.*At*3GT under *trc* promoter	This study
piBRTrc-glf.glk.pgm2.galU.*At*3GT.*Ph*ANS	piBRTrc vector carrying glf.glk.pgm2.galU under T7 promoter and *At*3GT.*Ph*ANS under *trc* promoter	This study
piBRUV5-glf.glk.pgm2.galU.*Ph*ANS.*At*3GT	piBRUV5 vector carrying glf.glk.pgm2.galU under T7 promoter and *Ph*ANS.*At*3GT under *lac*UV5 promoter	This study
piBRUV5-glf.glk.pgm2.galU.*At*3GT.*Ph*ANS	piBRUV5 vector carrying glf.glk.pgm2.galU under T7 promoter and *At*3GT.*Ph*ANS under *lac*UV5 promoter	This study

Strains	Description	Source/references
*Escherichia coli* XL-1 blue (MRF′)	General cloning host	Stratagene, USA
*E. coli* BL21 (DE3)	*ompT hsdT hsdS* (rB-mB-) gal (DE3)	Novagen, Germany
*E. coli* BL21 (DE3) piBR181.Apr	*E. coli* BL21 (DE3) carrying piBR181.Apr	This study
*E. coli* BL21 (DE3) piBRTrc.Apr	*E. coli* BL21 (DE3) carrying piBRTrc.Apr	This study
*E. coli* BL21 (DE3) piBRUV5.Apr	*E. coli* BL21 (DE3) carrying piBRUV5.Apr	This study
*E. coli* BL21 (DE3) piBR181.gfp	*E. coli* BL21 (DE3) carrying piBR181.gfp	This study
*E. coli* BL21 (DE3) piBRTrc.gfp	*E. coli* BL21 (DE3) carrying piBRTrc.gfp	This study
*E. coli* BL21 (DE3) piBRUV5.gfp	*E. coli* BL21 (DE3) carrying piBRUV5.gfp	This study
*E. coli* BL21 (DE3) piBRUV5-*Ph*ANS	*E. coli* BL21 (DE3) carrying piBRUV5-*Ph*ANS	This study
*E. coli* BL21 (DE3) piBRUV5-*At*3GT	*E. coli* BL21 (DE3) carrying piBRUV5-*At*3GT	This study
*E. coli* BL21 (DE3) piBR181-*Ph*ANS	*E. coli* BL21 (DE3) carrying piBR181-*Ph*ANS	This study
*E. coli BL21 (DE3)* piBR181-*At*3GT	*E. coli* BL21 (DE3) carrying piBR181-*Ph*ANS	This study
*E. coli* BL21 (DE3) piBRTrc-*Ph*ANS	*E. coli* BL21 (DE3) carrying piBRTrc-*Ph*ANS	This study
*E. coli* BL21 (DE3) piBRTrc-*At*3GT	*E. coli* BL21 (DE3) carrying piBRTrc-*At*3GT	This study
*E. coli* BL21 (DE3) piBRUV5-*Ph*ANS.*At*3GT (S_1_)	*E. coli* BL21 (DE3) carrying piBRUV5-*At*3GT.*Ph*ANS in monocistronic configuration	This study
*E. coli* BL21 (DE3) piBRUV5- *At*3GT. *Ph*ANS (S_2_)	*E. coli* BL21 (DE3) carrying piBRUV5-*At*3GT.*Ph*ANS in monocistronic configuration	This study
*E. coli* BL21 (DE3) piBR181-*Ph*ANS.*At*3GT (S_3_)	*E. coli* BL21 (DE3) carrying piBR181-*Ph*ANS.*At*3GT in monocistronic configuration	This study
*E. coli* BL21 (DE3) piBR181-*At*3GT. *Ph*ANS (S_4_)	*E. coli* BL21 (DE3) carrying piBR181-*At*3GT.*Ph*ANS in monocistronic configuration	This study
*E. coli* BL21 (DE3) piBRTrc-*Ph*ANS.*At*3GT (S_5_)	*E. coli* BL21 (DE3) carrying piBRTrc-*Ph*ANS.*At*3GT in monocistronic configuration	This study
*E. coli* BL21 (DE3) piBRTrc- *At*3GT. *Ph*ANS (S_6_)	*E. coli* BL21 (DE3) carrying piBRTrc-*At*3GT.*Ph*ANS in monocistronic configuration	This study
*E. coli* BL21 (DE3) piBRUV5-glf.glk.pgm2.galU.*Ph*ANS.*At*3GT (S_7_)	*E. coli* BL21 (DE3) carrying piBRUV5 vector carrying glf.glk.pgm2.galU under T7 promoter and *Ph*ANS.*At*3GT under *lac*UV5 promoter in monocistronic configuration	This study
*E. coli* BL21 (DE3) piBRUV5-glf.glk.pgm2.galU.*At*3GT.*Ph*ANS (S_8_)	*E. coli* BL21 (DE3) carrying piBRUV5 vector carrying glf.glk.pgm2.galU under T7 promoter and *At*3GT.*Ph*ANS under *lac*UV5 promoter in monocistronic configuration	This study
*E. coli* BL21 (DE3) piBR181-glf.glk.pgm2.galU.*Ph*ANS.*At*3GT (S_9_)	*E. coli* BL21 (DE3) carrying piBR181-glf.glk.pgm2.galU.*Ph*ANS.*At*3GT in monocistronic configuration	This study
*E. coli* BL21 (DE3) piBR181-glf.glk.pgm2.galU.*At*3GT.*Ph*ANS (S_10_)	*E. coli* BL21 (DE3) carrying piBR181-glf.glk.pgm2.galU.*At*3GT.*Ph*ANS in monocistronic configuration	This study
*E. coli* BL21 (DE3) piBRTrc-glf.glk.pgm2.galU.*Ph*ANS.*At*3GT (S_11_)	*E. coli* BL21 (DE3) carrying piBRTrc vector carrying glf.glk.pgm2.galU under T7 promoter and *Ph*ANS.*At*3GT under *trc* promoter in monocistronic configuration	This study
*E. coli* BL21 (DE3) piBRTrc-glf.glk.pgm2.galU.*At*3GT.*Ph*ANS (S_12_)	*E. coli* BL21 (DE3) carrying piBRTrc vector carrying glf.glk.pgm2.galU under T7 promoter and *At*3GT.*Ph*ANS under *trc* promoter in monocistronic configuration	This study

### Construction of UDP-d-glucose genes harboring C3G biosynthesis system

Based on the previous studies, the cytosolic pool of UDP-d-glucose is the limiting factor for the enhanced production of C3G [24, 25]. Thus, we included UDP-d-glucose pool enhancing biosynthesis pathway genes into the *Ph*ANS and *At*3GT harboring systems. The UDP-d-glucose system consisting of three biosynthesis genes: glucokinase (*glk)*, phosphoglucomutase (*pgm2*), and a glucose 1-phosphate uridylyltransferase (*galU*) for making UDP-d-glucose, along with a glucose facilitator diffusion protein (*glf*), cloned into piBR181 under P_T7_ promoter [31], was used to assemble plasmids with different combinations of *Ph*ANS and *At*3GT (Table 1, Additional file 1: Figure S6). The *glf* helps in the internalization of the externally added glucose, while the *glk* catalyzes the addition of phosphate groups into the 6-hydroxyl position of glucose immediately after entry into the cell, whereas the *pgm2* synthesizes glucose 1-phosphate from glucose-6-phosphate, and finally the *galU* transfers the uridine diphosphate (UDP) group to make UDP-d-glucose, thereby increasing the pool of UDP-d-glucose [31]. The vector physical map of both sugar cassettes, along with the assembly of genes in different combinations, is given in the supplementary data (Additional file 1: Figure S6).

### Production of cyanidin 3-*O*-glucoside, analysis and quantification

The biosynthesis of cyanidin and C3G was first carried out in the recombinant *E. coli* strains S_1_–S_6_ by harvesting the *E. coli* strain cultured in Luria Bertaini (LB) medium. The cells were prepared following 18 h of induction of recombinant proteins by isopropyl-*β*-d-thiogalactopyranoside (IPTG). The recombinant *E. coli* cells were harvested by centrifugation, and the thus obtained cell pellets were resuspended in the modified M9 minimal medium of pH 5, with a reduction in 1/5th of the initial volume at an OD_600_ ~ 15. To it, 1% sterile d-glucose was added, and the medium was supplemented with other components, such as glutamate and thiamine, 1 mM of (+)-catechin, 1 mM IPTG, 0.1 mM 2-oxoglutarate, 2.5 mM sodium ascorbate, and 50 μg/mL of kanamycin, as described in the methods section. The fermentation was then continued for 36 h.

**Fig. 3:**
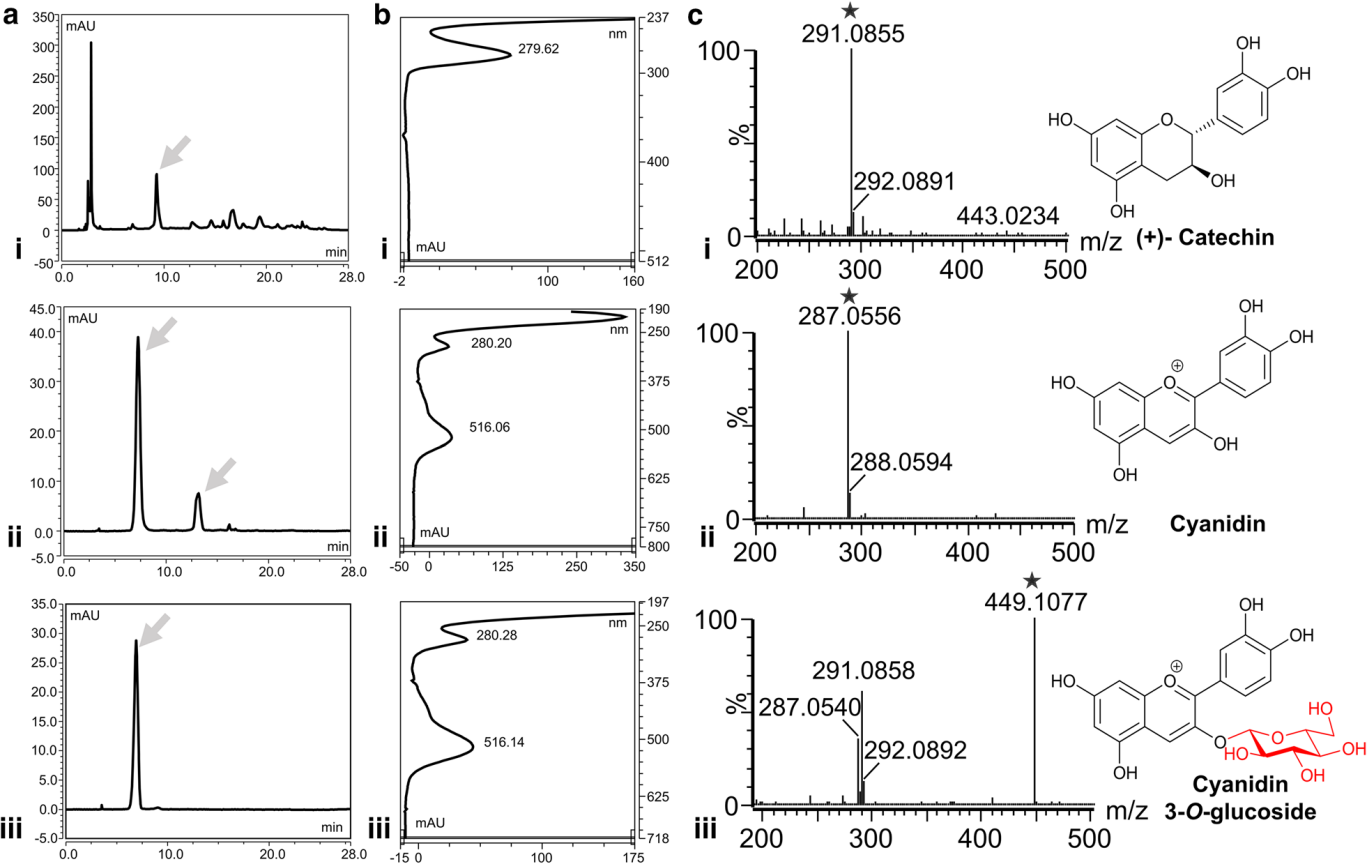
Spectroscopic analysis showing biosynthesis of cyanidin-3-*O*-glucoside (C3G) (**a**) UHPLC-PDA chromatogram, and **b** UV/VIS absorbance of (i) (+)- catechin, (ii) cyanidin and C3G, and (iii) C3G standard. **c** HR-QTOF-ESI/MS analysis confirming the production of C3G, along with the production of intermediate compound cyanidin (i) (+)-catechin, (ii) cyanidin, and (iii) C3G

The fermentation broth was centrifuged, and the presence of different metabolites was analyzed in both intracellular fraction and extracellular fraction. The UHPLC-PDA analysis of the sample showed three distinct peaks at the retention times (*t*_R_) of (9, 12.7, and 7.5) min, respectively (Fig. 3a(i, ii, iii)). A peak appearing at 9 min showing the UV maxima at ~ 280 nm was that of the substrate (+)-catechin (Fig. 3b(i)). The UV/VIS spectra showed the maximum absorbance of 516 nm for two peaks appearing at (12.7 and 7.5) min, which resemble the UV maxima of anthocyanidin compounds, such as cyanidin and C3G. Moreover, the standard C3G was used, in order to confirm the production of C3G from the engineered biosystem. The UHPLC-PDA analysis and absorbance of standard C3G exactly matched with the peak appearing at *t*_R_ 7.5 min (Fig. 3b(ii, iii)). Each peak was further analyzed by high-resolution quadruple time-of-flight electrospray ionization mass spectrometry (HR-QTOF-ESI/MS) in positive mode ionization, in order to identify their structure. The peak appearing at *t*_R_ 9 min showed a spectrum of m/z 291.0855, which resembles the calculated mass 291.0863 Da [C_15_H_15_O_6_^+^] of substrate (+)-catechin (Fig. 3c(i)), whereas the peak appearing at *t*_R_ 12.7 min showed a prominent spectrum m/z 287.0556 with 100% relative abundance, which resembles the calculated exact mass 287.0550 Da of cyanidin of molecular formula C_15_H_11_O_6_^+^ (Fig. 3c(ii)). Similarly, the peak appearing at *t*_R_ of 7.5 (Fig. 3a(ii)) showed a mass spectrum of m/z 449.1077, which matched with the mass of glucose conjugated cyanidin for which the exact calculated mass was 449.1078 Da [C_21_H_21_O_11_^+^]. An additional fragment of m/z 287.0540 of cyanidin also appeared, which differed from the exact mass of 162.0537 Da, representing the loss of glucose moiety from C3G (Fig. 3c(iii)). Figure 3c shows the structures of these compounds. This spectrometric evidence, along with ultra-high performance liquid chromatography-photo diode array (UHPLC-PDA) comparison with standard C3G, confirmed the production of cyanidin and C3G from the engineered system.

**Fig. 4:**
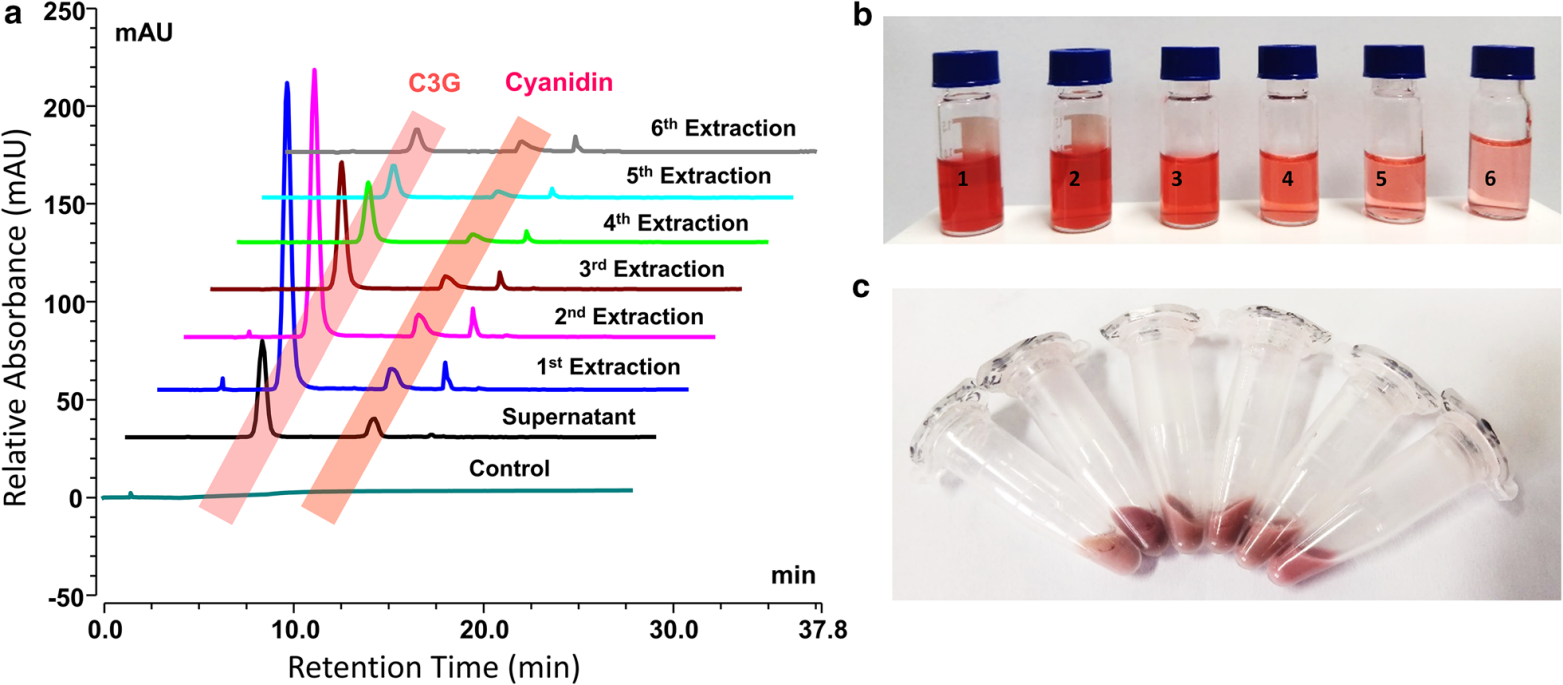
Isolation, extraction, and analysis of both extracellular and intracellular cyanidin-3-*O*-glucoside (C3G) in *E. coli* culture in modified M9 minimal medium after 36 h of incubation. **a** UHPLC-PDA profile of C3G. **b** Extraction of intracellular C3G by sonication, followed by centrifugation. The vials 1, 2, 3, 4, 5, and 6 show supernatant after each extraction. **c** The remaining cell pellets of different C3G producing strains after final extraction

After centrifugation of the fermentation broth, the cell pellets were extracted with 1% hydrochloric acid (HCl) in water for up to six times by sonication, as described in “Methods” section. The UHPLC-PDA analysis was done for the supernatant and all the extracted samples, which showed the production of C3G. An *E. coli* BL21 (DE3) strain without anthocyanin producing genes was taken as a control (Fig. 4a). The total amount of C3G titre was calculated on the basis of the peak area of the supernatant and the extracted samples. The concentration of the C3G decreased after each extraction with decrease in color intensity of the extracted sample in the HPLC vials (Fig. 4b). A very small amount of C3G along with the intermediate could be seen in the extracted cell pellet of different C3G producing strains after final extraction, as evidenced by the light shade of magenta retained by the cell (Fig. 4c).

### Production profiles of cyanidin and C3G in combinatorial engineered strains

**Fig. 5:**
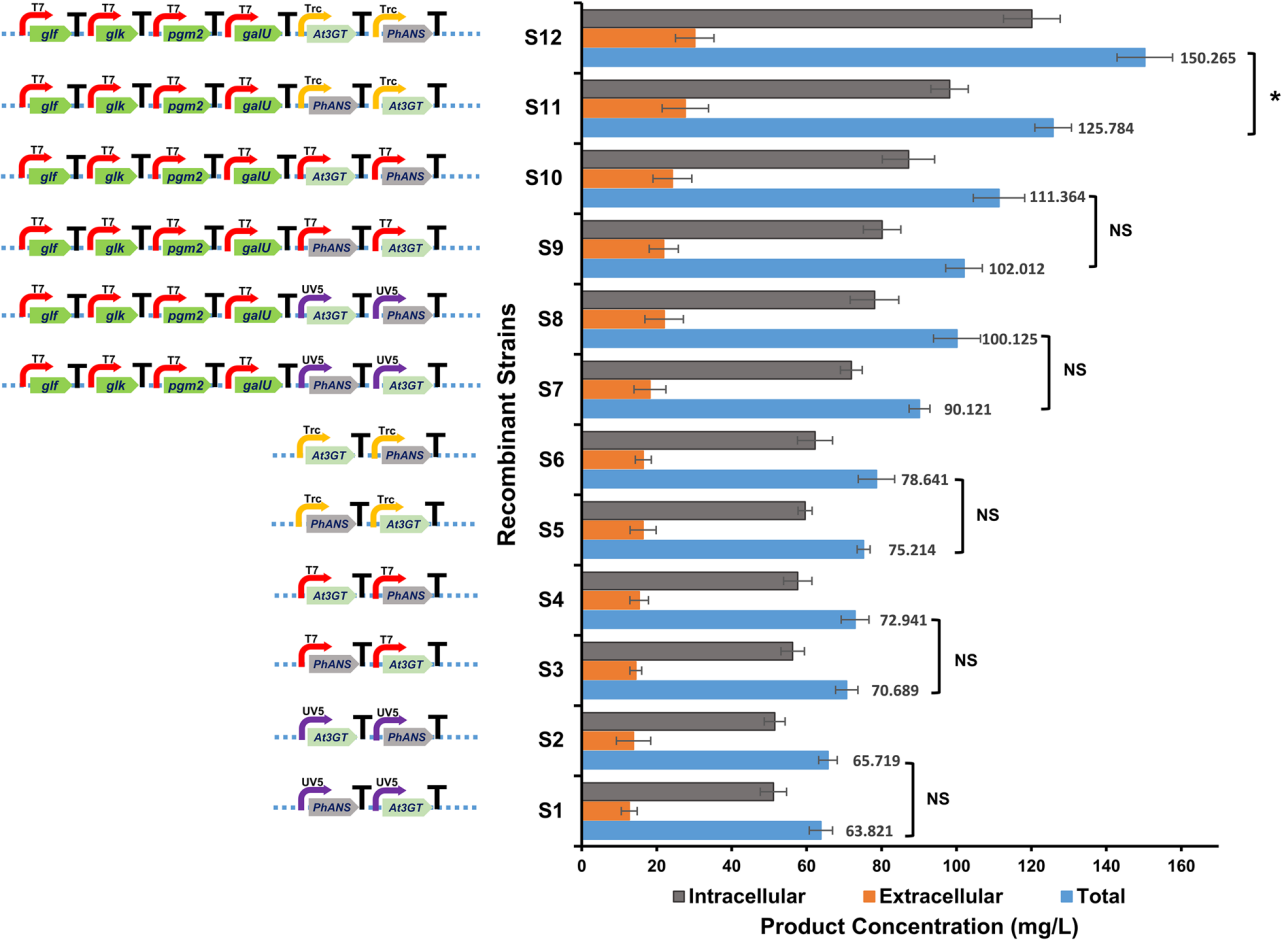
Cyanidin-3-*O*-glucoside (C3G) production comparison of twelve different recombinants showing the combinations of the genes, along with their respective promoters, in monocistronic fashion. Both the extracellular, intracellular and the total C3G titer of each strain has been shown. Error bars represent standard deviations. (NS) indicates not significant difference *p *> 0.05, and (*) denotes significant difference in C3G yield between the compared strain with *p *< 0.05

The production profiles of cyanidin and C3G were checked in all engineered strains (S_1_–S_12_) under the exactly identical conditions as described above. As anticipated, the production profiles of each strain varied from one another. The production profiles of the strains were slightly different when *At*3GT and *Ph*ANS were put forth and back under the same promoter. For example, the production profiles showed ~ 62 mg/L of C3G in S_1_ and ~ 68 mg/L in S_2_, which were both under the influence of P_*lac*UV5_ promoter. In the case of strains S_3_ and S_4_, which were under P_T7_ promoter, the C3G production was (~ 70 and ~ 79) mg/L, respectively, whereas in strains S_5_ and S_6_, the production increased to (~ 74 and ~ 85) mg/L, respectively, under the P_*trc*_ promoter (Fig. 5).

Previous studies by various groups have noted the availability of UDP-d-glucose as the rate-limiting factor for the increased C3G production, as the anthocyanin production occurs when the *At*3GT transfers glucose moiety from UDP-d-glucose to the 3-OH position of the intermediate cyanidin [22, 24, 25]. Keeping this in mind, the obvious choice for us was to overexpress the UDP-d-glucose biosynthesis genes with a glucose transporter gene. Thus, the UDP-d-glucose biosynthesis cassette was introduced into the system along with *At*3GT and *Ph*ANS, to generate the strains S_7_, S_8_, S_9_, S_10_, S_11_, and S_12_. These strains were checked for the production of C3G. The strains S_7_ and S_8_ with P_*lac*UV5_ promoter showed the C3G production of (90 and 100) mg/L, respectively, whereas in the strains S_9_ and S_10_ both under the P_T7_ promoter, the production reached (102 and 111) mg/L, respectively. Likewise, the strains S_11_ and S_12_ with P_*trc*_ promoter showed the C3G production of (125 and 150) mg/L, respectively. The C3G production increased in all these strains in which the UDP-d-glucose genes were over expressed; and among all strains, the strain S_12_ showed a noticeable improvement (Fig. 5).

### Glucose utilization and improved C3G production

**Fig. 6:**
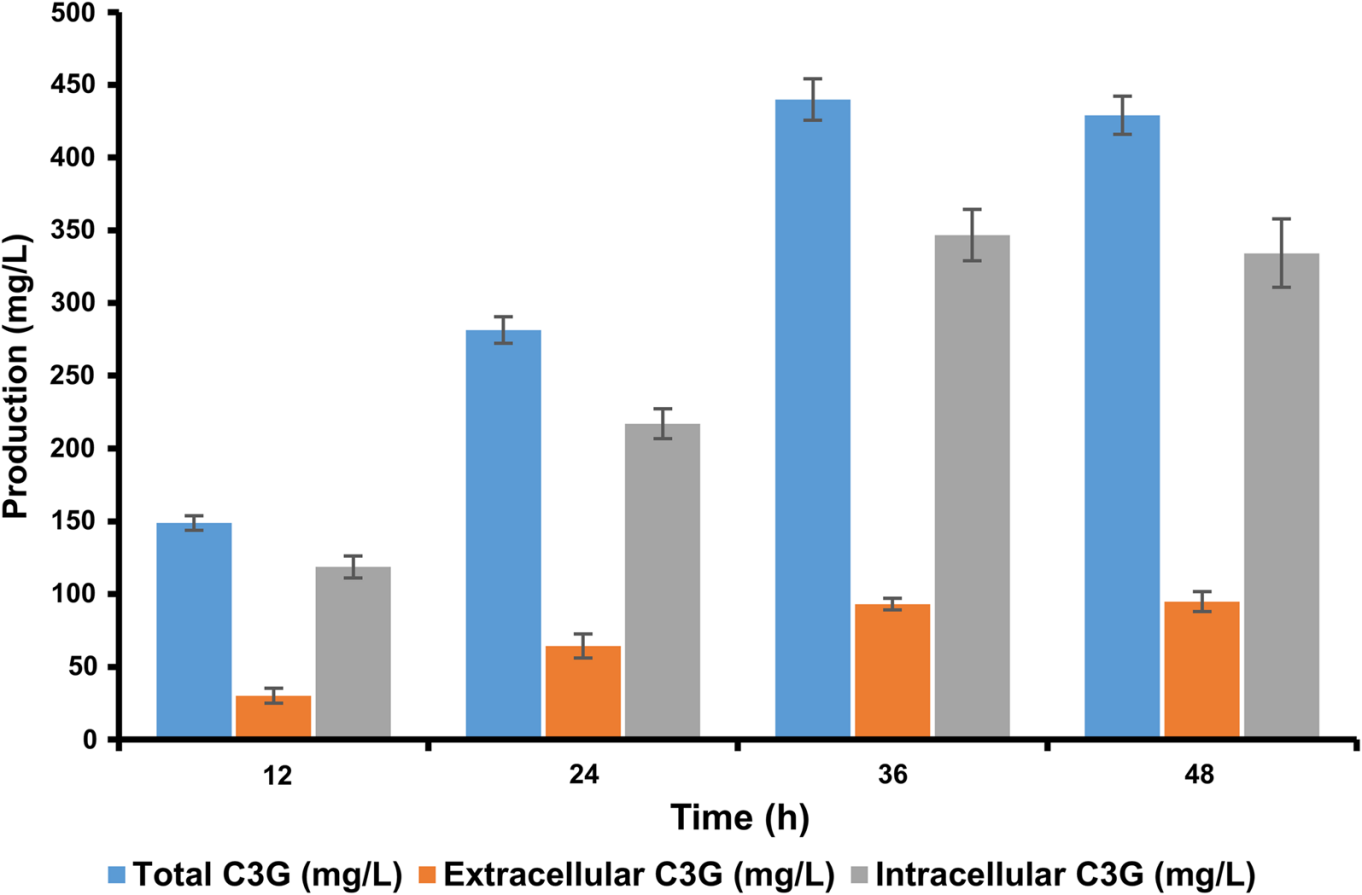
Time-dependent production profile of cyanidin-3-*O*-glucoside (C3G) using the recombinant strain S12 in 48 h cultivation time showing both extracellular, intracellular and the total C3G yield. The sample was taken at a 12 h time interval, and the recombinants were cultured in modified M9 minimal media. Error bars represent standard deviations

After checking the production of C3G in all twelve different strains, the strain S_12_ with piBRTrc-glf.glk.pgm2.galU.*At*3GT.*Ph*ANS plasmid, which initially gave the highest production of C3G, was chosen for further experimentation for the improvement of C3G production. The UDP-d-glucose expression cassette consisted of the *glf* gene, which is a glucose transporter gene, and facilitates the uptake of glucose inside the cell. Thus, three different concentrations of glucose (5, 10, and 15)% and a control (1% of glucose) were supplemented in production culture of the strain S_12_. The substrate (+)-catechin at a concentration of 2 mM was fed exogenously for the production of C3G, and the utilization of glucose was determined by the amount of C3G formation. The UHPLC-PDA analysis showed that at 5% glucose concentration, the C3G production reached its highest at ~ 439 mg/L after 36 h incubation time (Fig. 6).

**Fig. 7:**
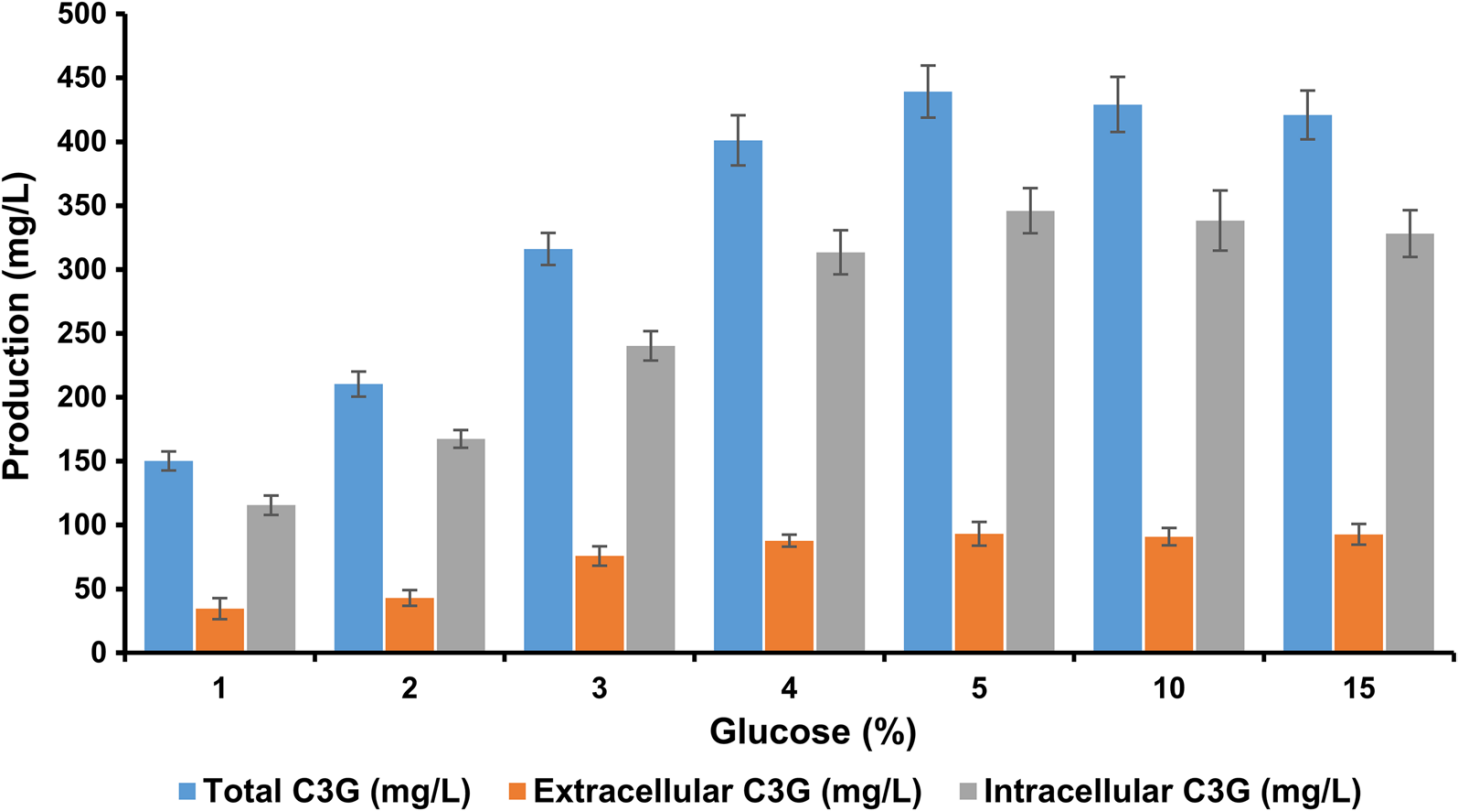
Effect of different concentration of glucose of (1, 2, 3, 4, 5, 10, and 15)% on cyanidin-3-*O*-glucoside (C3G) production using the recombinant strain S_12_ after 36 h of cultivation showing both extracellular, intracellular and the total C3G yield. Maximum production of C3G was achieved while 5% glucose was supplemented in the medium. Error bars represent standard deviations

Additionally, we also carried out the C3G production by varying the glucose gradient concentration between 1 and 5%, i.e. 1%, 2%, 3%, 4%, 5%. C3G production in 2%, 3% and 4% glucose was found to be ~ 210 mg/L, ~ 316 mg/L and ~ 401 mg/L respectively and found that the highest production of C3G was observed in 5% glucose concentration. Further increment in glucose concentration to (10 and 15)% did not increase the product formation, as the production remained almost the same at ~ 429 mg/L at 10% of glucose supplementation, and decreased to ~ 420 mg/L at 15% of glucose concentration at 36 h. Both the extracellular and the intracellular yield of C3G was determined and reported along with the total C3G yield. Thus, the use of the recombinant strain S_12_ for comparing the production profile under different glucose concentrations suggests that the presence of the glucose transporter protein along with the genes for the C3G biosynthesis gave the highest production when 5% glucose was fed exogenously with an increase of around 2.9-fold, when compared to the strain supplemented with only 1% glucose (Fig. 7).

## Discussion

Extensive studies have been carried out on the biosynthesis of plant-derived natural products like anthocyanins through the use of metabolic engineering approaches and synthetic biology tools. Anthocyanins are of particular interest, because of their wide application in the food and cosmetic industries and their health beneficial properties. However, their biosynthesis is complex as they are highly unstable compounds which makes it challenging for the commercial production of anthocyanins in controlled systems [32]. Various approaches have been applied previously for the increased production of C3G in *E. coli*, such as enhancing the substrate and precursor availability, optimizing the culture conditions, optimizing the expression level of the genes, and the use of the transporter proteins for the efflux of C3G from inside the cell [24, 25]. However, this study highlights the application of the combinatorial approach using a *Bgl*Brick multi-monocistronic vector system with promoters of varying strength. A total of six genes from C3G and UDP-d-glucose production were cloned into a single vector, and this system was harnessed for the efficient biosynthesis of C3G starting from (+)-catechin.

A multivariate multi-monocistronic operon system with three different promoters P_T7_, P_*trc*_, and P_*lac*UV5_ was used. Two new vectors piBRTrc and piBRUV5 were constructed, in order to clone multiple genes; each under their own promoter. Out of these three promoters, the P_T7_ was shown to be a stronger promoter than the P_*lac*UV5_ and P_*trc*_ promoters. The use of different promoters of different strength affects the amount of soluble protein obtained, and this in turn affects the production of the final compound [33]. In addition, by tuning the promoter strength and other factors like plasmid copy numbers, various key secondary metabolites, like artemisinin, taxol, and naringenin, have been produced by optimizing the biosynthetic pathways [34–36]. The use of a single vector system for assembly of multiple genes in a multi-monocistronic approach has proven beneficial over the obsolete strategies involving several vector system having different antibiotic resistance genes for the construction of microbial cell factories [37].

In order to validate whether the newly constructed vectors worked efficiently, two reporter genes *apr*^r^ and *gfp* were individually cloned in all the three vectors piBR181, piBRTrc, and piBRUV5, and the protein expression of the *apr*^r^ checked. The fluorescence of *gfp* was also checked using blue light emission (Fig. 2b). Next, the genes for anthocyanin production: *Ph*ANS and *At*3GT were first cloned individually in all of the three vectors, followed by in multi-monocistronic fashion. A total of eighteen different strains were constructed, all harboring the genes for anthocyanin production in *E. coli* BL21 (DE3). Out of these eighteen strains, twelve strains were checked for the production of C3G. The two genes *Ph*ANS and *At*3GT were shuffled back and forth under the same promoter, and their production was compared. The production results showed that when the *At*3GT gene was placed first during the cloning, the yield of C3G increased, compared to the other strains in which *Ph*ANS was placed first. These strains could accumulate cyanidin as intermediate, because of limited UDP-d-glucose in the cytosol. The enhanced production of C3G when At3GT was placed forward is still subject of investigation. However, the possible reasons could be stability of the mRNA transcript and expression of the genes even though they are cloned under individual promoters. The difference in production of C3G in all these strains was noteworthy when UDP-glucose pathway genes were co-expressed along with glucose facilitator gene. Out of the 12 strains whose production was checked, the strain S_12_ gave the highest titer of ~ 150 mg/L of C3G (Fig. 5). This strain was chosen for further production optimization and the enhancement of C3G production.

In a microbial system, glucose catabolism takes place through glycolysis and the pentose phosphate pathway. These pathways not only provide the energy for cell growth and other small molecules, but also support anabolic activities, which can further be used for the microbial synthesis of different natural products through different biosynthetic pathways and reactions [38]. Thus, the use of glucose exceeds not only in catabolism uses, but also as a carbon source for the biosynthesis of the structural units of the different glycosylated molecules necessary for microorganisms. Most of the metabolic engineering strategies involving NDP-sugar involves diversion of the metabolic flux either by deletion or overexpression of pathway specific genes so that there is an increase in the level of the desired sugar [39]. However, the deletion of genes and the pathway diversion tend to require additional reinforcement of the carbon source, thereby limiting the optimal growth of the desired strain.

As evident from the previous studies [24, 25], the availability of the glycosyl donor, UDP-d-glucose, is the key bottleneck in the production of C3G. Thus, when they overexpressed the UDP-d-glucose biosynthesis genes along with the C3G biosynthesis genes, the production was reported at around 120 mg/L, and with optimization in culture conditions, induction optimization, and the fermentation approach, the production reached 350 mg/L [25]. However, our study incorporated a strategy that involved the overexpression of the genes for UDP-d-glucose biosynthesis, along with a glucose transporter proteins *glf* that was responsible for the uptake of exogenously-fed glucose to inside the *E. coli* cytoplasm [40], and the enzymes *glk*, *pgm,* and *galU* redirected the flux of intracellular glucose to UDP-d-glucose synthesis [39]. Although several glucose transporter genes are known in *E. coli* system, we choose *glf*-*glk* system for this study as it is well-characterized and readily available in our lab. Additionally, the working efficiency of this system has been already demonstrated to produce plant polyphenol glycosides and other polyketides in the previous studies [31, 40, 41]. This system increased UDP-d-glucose production, thereby increasing the donor pool for subsequent glycosylation by the *At*3GT gene to produce C3G. The culmination of all this resulted in the highest production of C3G to ~ 439 mg/L, when 5% d-glucose and 2 mM (+)-catechin was fed using the strain S_12_. We also checked the time-dependent production of C3G, and identified the optimum production at 36 h (Fig. 6). The possible reason for enhanced production of C3G in the strains with *Ph*ANS and *At*3GT under P_*trc*_ promoter could be because of the optimum amount of proteins production in the cell. Most importantly, the cell health is maintained as significant amount of cell energy is utilized when proteins are expressed under strong promoter such as T7. This generally leads to reduced cell growth when multiple genes are co-expressed under P_*T7*_ promoter.

A standard *Bgl*Bricks protocol addresses the scar translation issue associated with BioBrick assembly. It employs *Bgl*II and *Bam*HI restriction enzymes resulting in a six nucleotides scar sequence encoding glycine-serine, a peptide linker in most fusion proteins. In our approach, we have minimized such bottlenecks of BioBrick and have taken advantage of *Bgl*Bricks [27] for efficient construction of multi-monocistronic genetic circuit. Our approach helps to excise all the bioparts at once and facilitates engineering the genetic circuit. It demonstrates the bio-parts available in a commercial vector can also serve as a valuable resource for initial synthetic biology that can be further refined to be successfully applied for biotechnological significance. Importantly, we designed vectors with two different promoters P_*lac*UV5_ and P_*trc*_ each having the same set of restriction sites which makes the bioparts compatible and interchangeable.

We successfully completed the design-build-test cycle [42] for the enhanced production of C3G in engineered *E. coli*. We designed the bio parts computationally, followed by the build stage, which began with commercial DNA synthesis, followed by digestion and ligation reactions into the vector backbone, in order to generate the suitable plasmids. Functionality assays of the newly constructed systems were also carried out via protein expression. Finally, in order to test the constructs, the newly obtained recombinants that includes C3G synthesis genes were checked for C3G production. Thus, the system efficiently produced a high titer of C3G, with 1.25-fold increase in production, compared to the previously reported titer [25]. However, it must be kept under consideration that the actual amount of C3G produced could be higher than our reported value, as evident from the figure, where even after the final extraction, the cell pellet retained the color because of remaining cyanidin and C3G inside the cytoplasm.

## Methods

### Chemicals and enzymes

(+)-Catechin was purchased from Sigma-Aldrich (St. Louis, MO, USA). Cyanidin 3-*O*-glucoside standard was purchased from Polyphenols (Sandnes, Norway). All restriction enzymes used in the cloning process were obtained from Takara (Shiga, Japan). Genes were synthesized from GenScript^®^ (Piscataway, NJ, USA), while oligonucleotide primers were synthesized from Genotech (Daejeon, Korea). High-performance liquid chromatography (HPLC) grade acetonitrile and water were purchased from Mallinckrodt Baker (Phillipsburg, NJ, USA). All other chemicals used were of high analytical grades, and were commercially available.

### Bacterial strains and culture conditions

Table 1 provides a list of all *Escherichia coli* strains and plasmids constructed in this work. Plasmid construction and propagation were performed in *E. coli* XL1-Blue. For the selection and maintenance of plasmids, *E. coli* strains were grown at 37 °C in Luria–Bertani (LB) broth, or on agar plates supplemented with an appropriate amount of antibiotics (kanamycin-50 μg/mL). All anthocyanin production experiments were performed using *E. coli* BL21 (DE3) as a host. The recombinant *E. coli* BL21 (DE3) with plasmids were first streaked into the LB agar plates containing appropriate antibiotics, and incubated overnight at 37 °C. The cells were then inoculated into a 15 mL tube containing 5 mL LB broth with kanamycin. After incubation for 3 h, 1 mL of the preinoculum was transferred into the 500 mL conical flask containing 50 mL of LB medium with antibiotics, and grown at 37 °C with shaking, followed by 1 mM IPTG induction at the optical density of ~ 0.8 at OD_600_. Protein expression was allowed for 18 h at 20 °C. All production experiments were performed in triplicate.

### Plasmids

Two different bio-parts were designed and synthesized, in order to generate two different multi-mono-cistronic vectors sharing similar cloning sites but different promoters (P_*trc*_ and P_*lac*UV5_). Commercially available pET-28a (+) vector was modified to a piBRTrc and piBRUV5 expression vector using a synthetic dsDNA construct. Previously constructed piBR181 was also used for cloning purposes. The genes *Ph*ANS and *At*3GT were synthesized from Genescript^®^ (Piscataway, NJ, USA). The genes were then excised using the restriction sites *Xba*I/*Hin*dIII, and ligated individually into each of the expression vectors piBR181, piBRTrc, and piBRUV5-*At*3GT, and *Ph*ANS were then assembled into monocistronic operon configurations with each gene flanked by its own promoter and terminator, followed by ligation of restriction digestion fragments from the plasmid. Table 1 provides details of the expression and sub-cloning plasmids, as well as the strains used in this study.

### Functionality assay of the newly constructed vector

An apramycin acetyltransferase resistance gene (*apr*^r^) from the pKC1139 [43] and green fluorescent protein (*gfp*) expression gene was PCR amplified using the primer sets given in Additional file 1: Table S1. Both genes were cloned into the newly constructed vector system piBRTrc and piBRUV5, along with piBR181 (for comparison purposes). The amplification conditions for PCR were: 95 °C for 7 min, followed by 30 cycles at 95 °C for 1 min, 55 °C for 1 min, 72 °C for 1 min, and finally 72 °C for 7 min in a total volume of 50 μL. Following routine manipulation of the PCR products in pGEM^®^-T Easy vector, the *apr*^r^ and *gfp* were digested with restriction enzyme *Xba*I/*Hin*dIII. They were ligated individually to piBR181, piBRTrc, and piBRUV5 expression vectors at *Spe*I/*Hin*dIII sites, in order to generate the plasmids piBR181-Apr, piBRTrc-Apr, piBRUV5-Apr, piBR181-gfp, piBRTrc-gfp, and piBRUV5-gfp.

### Expression of *apr*^r^

The recombinant *E. coli* BL21 (DE3) harboring *apr*^r^ in piBR181, piBRTrc, and piBRUV5 were cultured in 50 mL LB media and incubated at 37 °C, until the optical density at 600 nm (OD_600_) reached ~ 0.8. Induction was carried out with the final concentration of 1 mM IPTG, and the culture was then further incubated at 20 °C for 18 h. Cells were harvested by centrifugation at 4 °C for 10 min at 842×*g*, and washed twice with 50 mM Tris–HCl buffer (pH 7.5) containing 10% glycerol. The final cell pellet was re-suspended in 1 mL of the Tris–HCl buffer, and lysed by ultra-sonication in ice bath. Then, cell debris was removed by centrifugation at 13,475 for 30 min at 4 °C. Finally, the obtained soluble protein in the supernatant was analyzed by 12% sodium dodecyl sulfate polyacrylamide gel electrophoresis (SDS-PAGE).

### Construction of plasmids for cyanidin 3-*O*-glucoside production

Table 1 lists all of the strains, vectors, and plasmids generated and used in this study. *Ph*ANS and *At*3GT gene were synthesized and cloned in the pUC57 vector. For the cloning of both genes into piBR181, piBRTrc, and piBRUV5 vectors, the genes were digested with the *Xba*I/*Hin*dIII restriction enzymes. piBR181, piBRTrc, and piBRUV5 were digested with the *Spe*I/*Hin*dIII enzymes. The digested amplicons and backbones were purified through gel electrophoresis, and individually ligated using T_4_ DNA ligase (Promega, USA), in order to construct the recombinants listed in Table 1. The ligation of the genes with the vectors resulted in the “scar” formation at the *Spe*I-*Hin*dIII. The ligated products were transformed into *E. coli* XL-1 Blue, and confirmed by restriction digestion.

*At*3GT and *Ph*ANS were then assembled into monocistronic configurations by ligation of the restriction digestion fragments from piBR181-*Ph*ANS (*Bam*HI/*Xho*I) and piBR181-*At*3GT (*Bgl*II/*Xho*I), and yielded plasmid piBR181-*Ph*ANS.*At*3GT, leaving the scar at the *Bam*HI-*Bgl*II sites. In addition, the ligation of restriction digestion fragments from piBR181-*Ph*ANS (*Bgl*II/*Xho*I) and piBR181-*At*3GT (*Bam*HI/*Xho*I) yielded plasmid piBR-*At*3GT.*Ph*ANS in a multi-monocistronic configuration. Similarly, the ligation of restriction digestion fragments from piBRTrc-*Ph*ANS (*Bam*HI/*Xho*I) and piBRTrc-*At*3GT (*Bgl*II/*Xho*I) yielded plasmid piBRTrc-*Ph*ANS.*At*3GT, and the ligation of restriction digestion fragments from piBRTrc-*Ph*ANS (*Bgl*II/*Xho*I) and piBRTrc-*At*3GT (*Bam*HI/*Xho*I) yielded plasmid piBRTrc-*At*3GT.PhANS. Finally, plasmid piBRUV5-*Ph*ANS.*At*3GT and piBRUV5-*At*3GT.*Ph*ANS were generated by ligating the restriction fragments from piBRUV5-*Ph*ANS (*Bam*HI/*Xho*I) and piBRUV5-*At*3GT (*Bgl*II/*Xho*I), and piBRUV5-*Ph*ANS (*Bgl*II/*Xho*I) and piBRUV5-*At*3GT (*Bam*HI/*Xho*I), respectively. This process can be iterated successively, in order to assemble an arbitrary number of parts together, repetitively using the same protocol.

Next, piBR181-glf.glk.pgm2.galU plasmid (UDP-d-glucose cassette harboring each genes under P_T7_ promoter) from a previous study was used as the backbone for assembling the *Ph*ANS and *At*3GT genes that were cloned earlier, in order to generate the plasmids. The *Bgl*II/*Xho*I digested DNA fragments from piBR181-*Ph*ANS.*At*3GT, piBR-*At*3GT.*Ph*ANS, piBRTrc-*Ph*ANS.*At*3GT, piBRTrc-*At*3GT.*Ph*ANS, piBRUV5-*Ph*ANS.*At*3GT, and piBRTrc-*At*3GT.P*h*ANS were individually ligated with *Bam*HI/*Xho*I digested piBR181-glf.glk.pgm2.galU recombinant plasmid, in order to generate piBR181-glf.glk.pgm2.galU.*Ph*ANS.*At*3GT, piBR181-glf.glk.pgm2.galU.*At*3GT.*Ph*ANS, piBRTrc-glf.glk.pgm2.galU.*Ph*ANS.*At*3GT, piBRTrc-glf.glk.pgm2.galU.*At*3GT.*Ph*ANS, piBRUV5-glf.glk.pgm2.galU.*Ph*ANS.*At*3GT, and piBRUV5-glf.glk.pgm2.galU.*At*3GT.*Ph*ANS, respectively. In these plasmids, the *glf*, *glk*, *pgm2*, and *galU* genes are expressed under the individual P_T7_ promoter, while *Ph*ANS and *At*3GT are expressed under either the P_T7_, P_*trc*_, or P_*lac*UV5_ promoters. These plasmids were transformed into the *E. coli* BL21 (DE3).

### Flask experiments and fermentation

*Escherichia coli* BL21 (DE3) cells harboring *Ph*ANS and *At*3GT were cultured in 500 mL *E. coli* shake flasks at 37 °C and at OD_600_ of ~ 0.8, then induced by 1 mM IPTG, and incubated at 20 °C for 18 h. Next, the culture was transferred to a 50 mL sterile conical tube, and centrifuged at 842×*g* at 4 °C for 15 min. The supernatant was then discarded, and the pellet was suspended in 10 mL modified M9 medium consisting of M9 salts (Na_2_HPO_4_, KH_2_PO_4_, NaCl, and NH_4_Cl), 0.4% glucose, 1 mM MgSO_4_, 0.1 mM CaCl_2_, and 2 × 10^−4^% thiamine, 2 × 10^−4^% glutamate, and pH adjusted to 5.0 by the addition of HCl. Cofactors like 2-oxoglutarate (0.1 mM) and sodium l-ascorbate (2.5 mM) were also added to the modified M9 media. Finally, the resuspended culture was fed with the substrate 2 mM (+)-catechin, 1 mM IPTG, and 50 μg/mL of kanamycin, and incubated at 20 °C for 36 h, prior to its analysis for C3G production. The culture was fed with 1% glucose after 3 h of incubation, in order to increase the UDP-d-glucose concentration in the cell for glycosylation.

### Extraction and analytical methods

#### Extraction of C3G

After 36 h of incubation, the colorless cell culture turned into a magenta color, which was caused by the production of cyanidin and C3G. The cultures from fermentation were first acidified with 1% HCl, and centrifuged at 842×*g* at 4 °C for 15 min, after which the supernatant and pellet were separated. The supernatant, which accounts for the extracellular C3G production, was collected in another tube, and to the remaining cell pellet, 1 mL water with 1% HCl was added, mixed well, and subjected to sonication for the extraction of the intracellular C3G from the cell of recombinant *E. coli*. Following sonication for 10 min, the content was centrifuged at 13,475×g for 5 min, and the supernatant and cell pellet were separated. The supernatant was isolated, and the extraction was repeated for the remaining cell pellet up to the sixth elution. After each elution, the sample was subjected to further UHPLC-PDA analysis.

#### UHPLC analysis

The cell-free culture medium obtained from fermentations and the extracted compound were analyzed by UHPLC-PDA, using a Thermo Scientific Dionex Ultimate 3000 ultrahigh-performance Liquid chromatography (UHPLC) system with a reverse-phase C18 column (Mightysil RP-18 GP) (4.6 mm × 250 mm, 5 μm particle size) (Kanto Chemical, Japan). The solvents used were HPLC-grade water and acetonitrile both containing 0.1% (v/v) formic acid as the mobile phases at a flow rate of 1.0 mL/min for 28 min. The elution protocol was as follows (acetonitrile concentration v/v): beginning with 10%, a linear gradient from (10 to 40%) for 10 min, 40–60% for 5 min, and 60–10% for 2 min, followed by washing and equilibration of the column. The absorbance was measured at 520 nm. The production of C3G was confirmed by comparing the UHPLC profile of the standard C3G with that of the extracted product. C3G was quantified by the standard curve obtained from the UHPLC-PDA profile of C3G injected at different concentrations of 5–200 μg/mL at the wavelength of 520 nm. The total production of C3G was determined based on the linear regression equation obtained from the standard curve. Furthermore, the production of C3G was characterized by HR-QTOF-ESI/MS [ACQUITY (UPLC, Waters, Milford, MA)-SYNAPT G2-S (Waters)] in positive ion mode.

## Additional file

**Additional file 1: Table S1.** Primers used in this study. **Figure S1.** piBRTrc *Bgl*Brick 382 bp vector sequence showing the *trc* promoter, *lac* operator, ribosome binding site and a transcriptional terminator along with the five different restriction sites. **Figure S2.** piBRUV5 *Bgl*Brick 172 bp vector sequence showing the *lac*UV5 promoter, *lac* operator, ribosome binding site and a transcriptional terminator along with the five different restriction sites. **Figure S3.** Construction of reporter genes for functionality assay of newly designed vectors (**a**) Apramycin resistance gene (*apr*^*r*^) were cloned separately into piBR181, piBRTrc and piBRUV5 generating different recombinants piBR181-Apr, piBRTrc-Apr, and piBRUV5-Apr respectively (**b**) GFP gene (*gfp*) were also cloned separately into piBR181, piBRTrc, and piBRUV5 generating piBR181-gfp, piBRTrc-gfp and piBRUV5-gfp respectively. **Figure S4.** Functionality assay of newly constructed vector system. Kanamycin and apramycin antibiotics susceptibility test in *E. coli* BL21 (DE3) harboring pET28a (+), piBR181, piBRTrc, and piBRUV5. Results shows a clear zone of inhibition in *E. coli* BL21 (DE3) in both antibiotics kanamycin and apramycin. In case of pET28a (+) with kanamycin resistance gene, the zone of inhibition is visible only in presence of apramcyin whereas zone of inhibition is absent in both antibiotics in piBR181-Apr, piBRTrc-Apr and piBRUV5-Apr harboring *E. coli* BL21 (DE3). **Figure S5.** Cloning strategy for the construction of a recombinant cyanidin-3-*O*-glucoside (C3G) production cassette in piBRTrc, piBRUV5, and piBR181 multi-monocistronicvector. Two genes anthocyanidin synthase (*Ph*ANS) and flavonol-3-*O*-glycosyltransferase (*At*3GT) were cloned together to generate the strains S_1_, S_2_, S_3_, S_4_, S_5_, and S_6_. **Figure S6.** Final assembly of cyanidin-3-*O*-glucoside system using the previously constructed UDP-d-glucose biosynthesis system in piBR181 to which the anthocyanidin synthase (*Ph*ANS) and flavonol-3-*O*-glycosyltransferase (*At*3GT) were further assembled in multi-monocistronic fashion in various combinations to generate the strains S_7_, S_8_, S_9_, S_10_, S_11_ and S_12_.

## Abbreviations

C3G: cyanidin 3-*O*-glucoside
UDP: uridine diphosphate
RBS: ribosome-binding site
GFP: green fluorescence protein
LB: Luria Bertaini
HPLC: high-performance liquid chromatography
UHPLC-PDA: ultra-high performance liquid chromatography-photo diode array
IPTG: isopropyl-*β*-d-thiogalactopyranoside
HR-QTOF-ESI/MS: high-resolution quadruple time-of-flight electrospray ionization mass spectrometry
