# Supplementary material for: Combinatorial approach for improved cyanidin 3-O-glucoside production in Escherichia coli
Source: Microb Cell Fact. 2019 Jan 17;18:7. doi: 10.1186/s12934-019-1056-6 (PMC6335687; doi:10.1186/s12934-019-1056-6)

Additional file 1

**Combinatorial Approach for Improved Cyanidin 3-*O*-Glucoside Production in *Escherichia coli***

**Biplav Shrestha^1,‡^, Ramesh Prasad Pandey^1,2,‡^, Sumangala Darsandhari^1^, Prakash Parajuli^1^, Jae Kyung Sohng^1,2,*^**

*^1^ Department of Life Science and Biochemical Engineering, SunMoon University, 70 Sunmoon-ro 221, Tangjeong-myeon, Asan-si, Chungnam 31460, Republic of Korea*

*^2^Department of BT-Convergent Pharmaceutical Engineering, SunMoon University, 70 Sunmoon-ro 221, Tangjeong-myeon, Asan-si, Chungnam 31460, Republic of Korea.*

Biplav Shrestha: biepluv@gmail.com

Ramesh Prasad Pandey: ramesh.pandey25@gmail.com

Sumangala Darsandhari: dsumanng@gmail.com

Prakash Parajuli: parajuli1985@gmail.com

Jae Kyung Sohng: sohng@sunmoon.ac.kr

***Corresponding author:**

Prof. Jae Kyung Sohng

Tel: +82(41)530-2246

Fax: +82(41)530-8229

^‡^ Authors contributed equally to this work.

**Running Title**: Improved Cyanidin 3-*O*-Glucoside Production

**Table S**1. Primers used in this study

| **Bacterial strains and plasmids** | **Genes** | **Primer (5′ - 3′)** |
| --- | --- | --- |
| pKC1139 plasmid | apr^r^ (777 bp) | F:TCTAGAATGCAATACGAATGGCGAAAAGCC(*Xba*I)  R:AAGCTTTCAGCCAATCGACTGGCGAGCGGC (*Hin*dIII) |
| Green fluroscent protein | gfp (717 bp) | F-TCTAGAATGAGTAAAGGAGAAGAA (*Xba*I)  R-AAGCTTCTATTTGTATAGTTCATCCAT-( *Hin*dIII) |

Figure S1


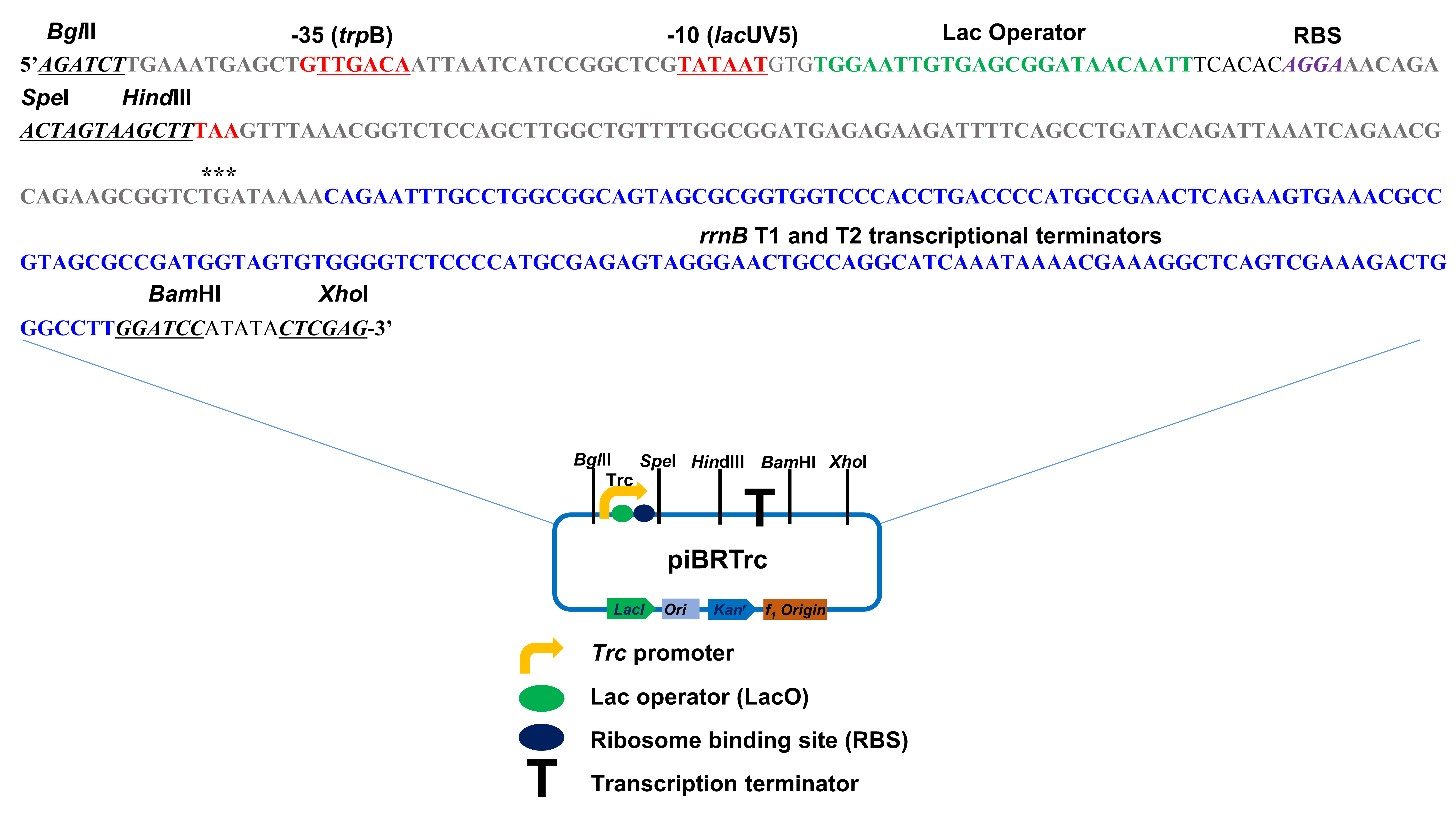


Figure S2


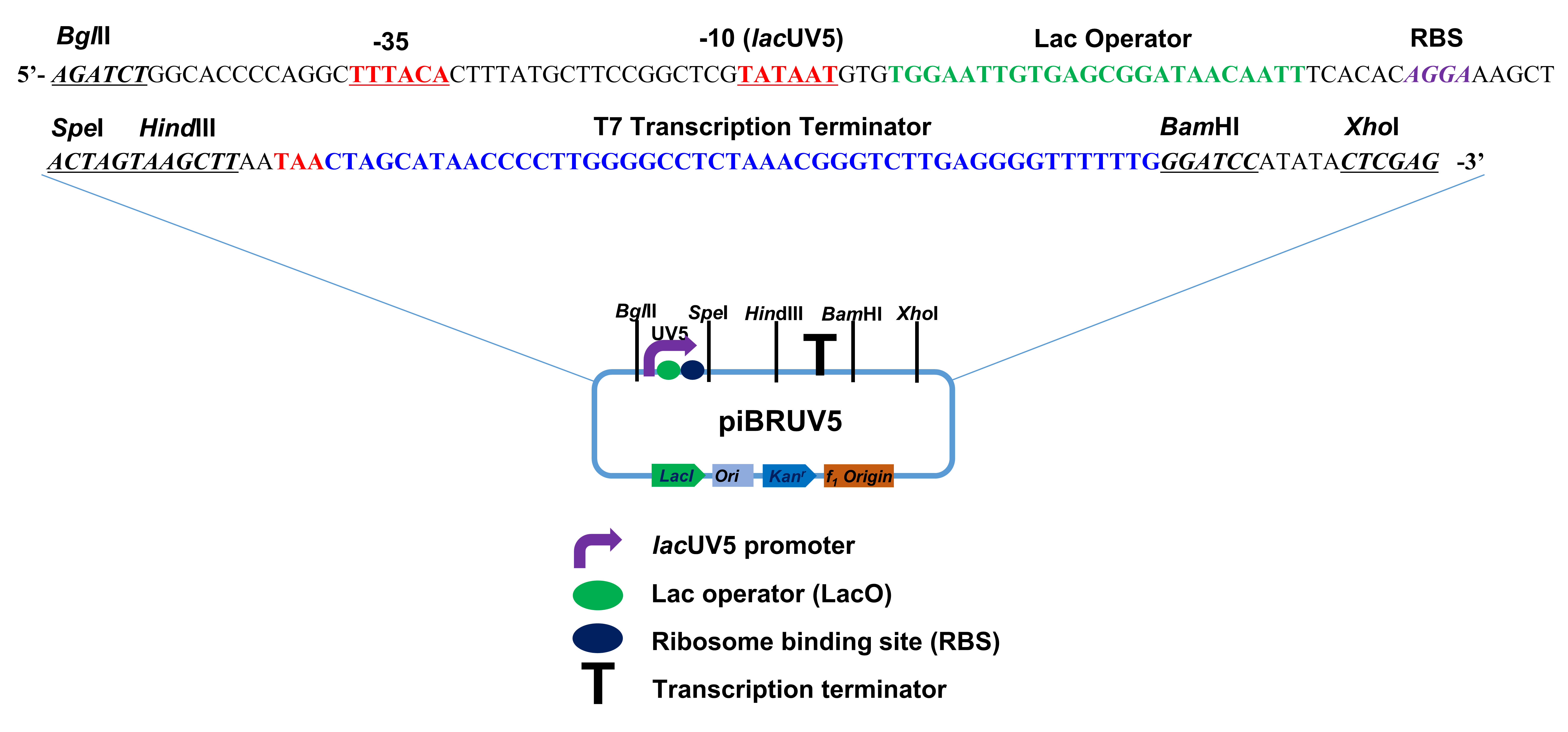


Figure S3


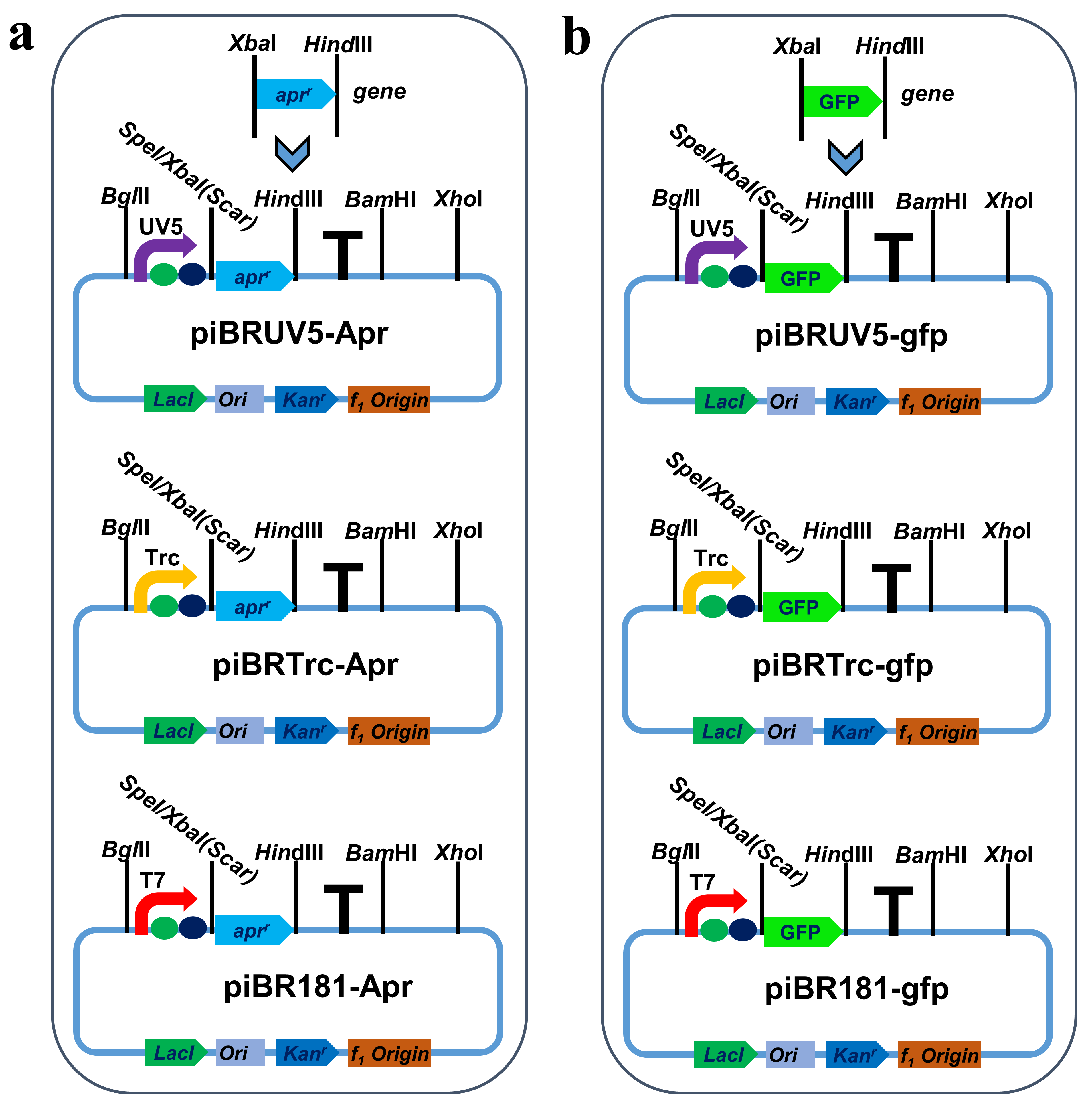


Figure S4


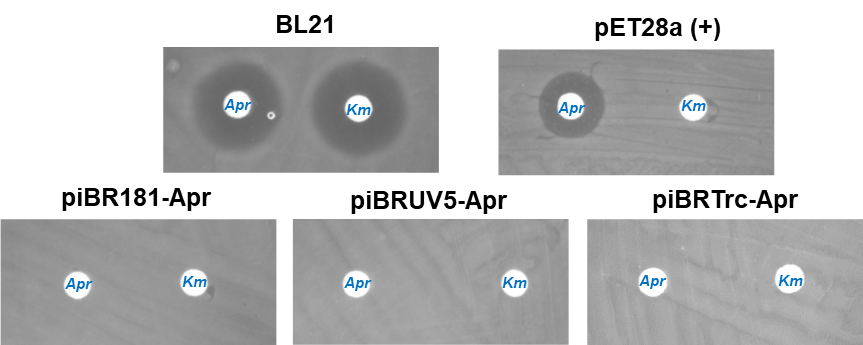


Figure S5


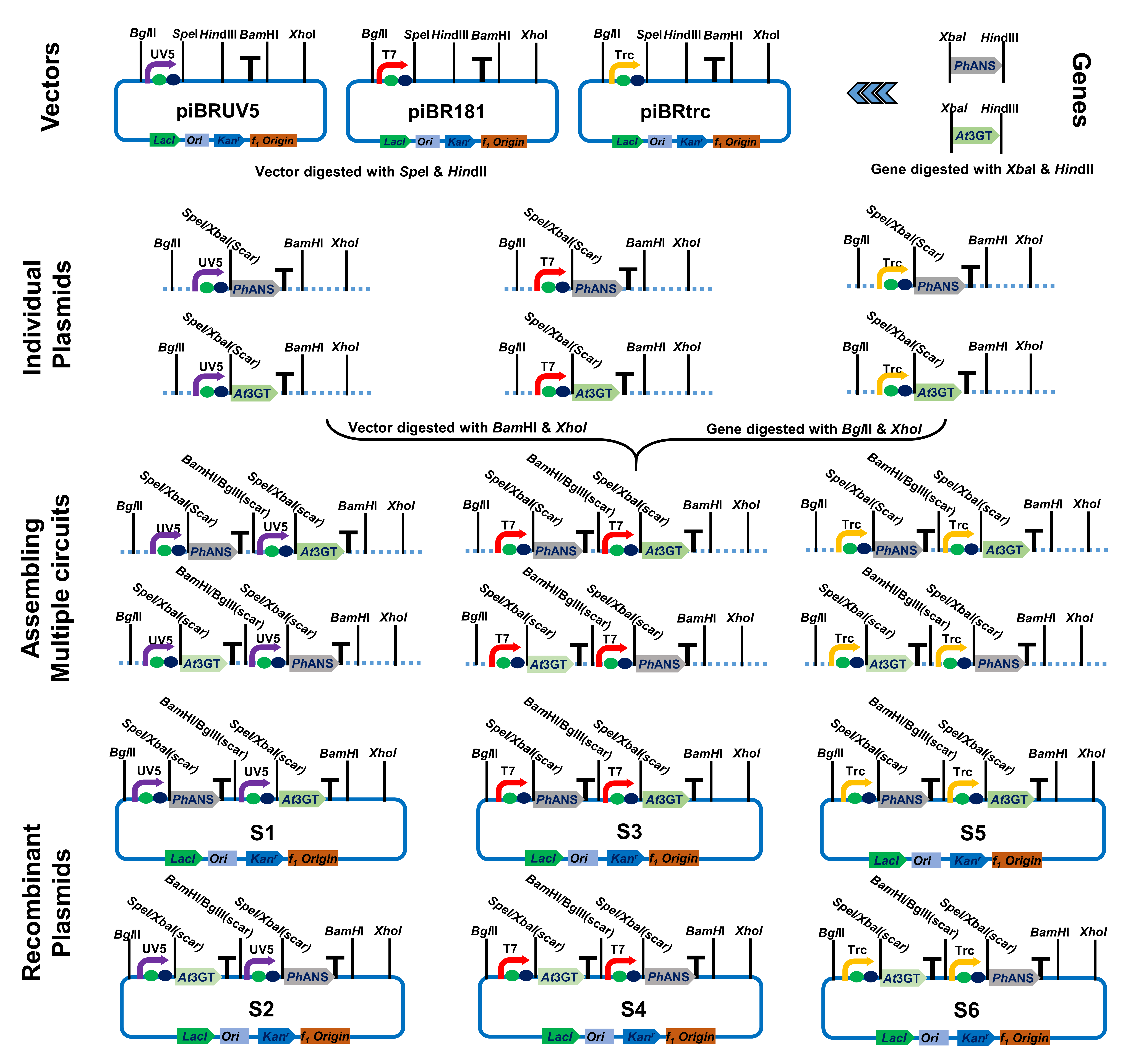


Figure S6


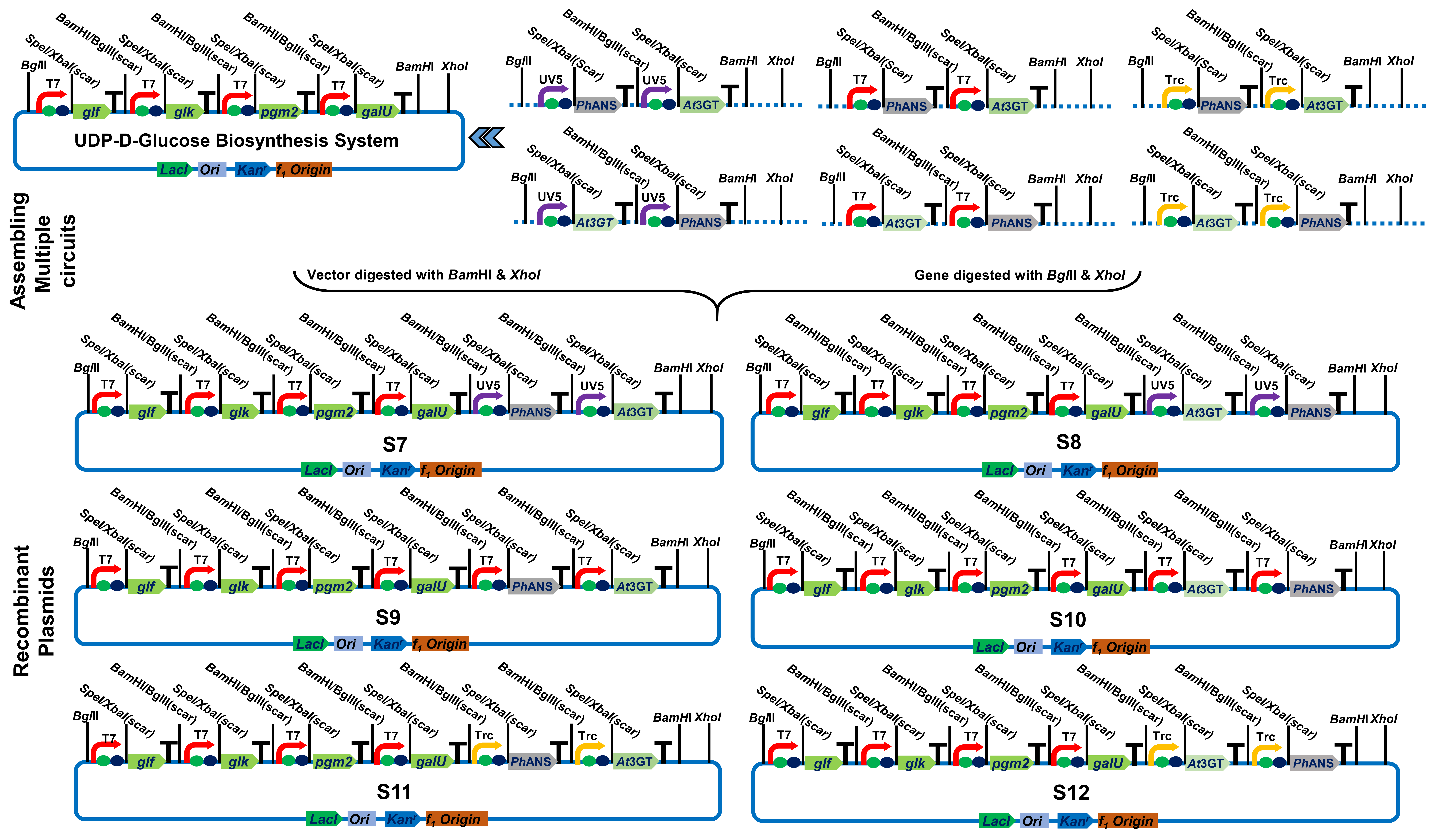

Supplement: Supplementary file 1 — Additional file 1: Table S1. Primers used in this study. Figure S1. piBRTrc BglBrick 382 bp vector sequence showing the trc promoter, lac operator, ribosome binding site and a transcriptional terminator along with the five different restriction sites. Figure S2. piBRUV5 BglBrick 172 bp vector sequence showing the lacUV5 promoter, lac operator, ribosome binding site and a transcriptional terminator along with the five different restriction sites. Figure S3. Construction of reporter genes for functionality assay of newly designed vectors (a) Apramycin resistance gene (aprr) were cloned separately into piBR181, piBRTrc and piBRUV5 generating different recombinants piBR181-Apr, piBRTrc-Apr, and piBRUV5-Apr respectively (b) GFP gene (gfp) were also cloned separately into piBR181, piBRTrc, and piBRUV5 generating piBR181-gfp, piBRTrc-gfp and piBRUV5-gfp respectively. Figure S4. Functionality assay of newly constructed vector system. Kanamycin and apramycin antibiotics susceptibility test in E. coli BL21 (DE3) harboring pET28a (+), piBR181, piBRTrc, and piBRUV5. Results shows a clear zone of inhibition in E. coli BL21 (DE3) in both antibiotics kanamycin and apramycin. In case of pET28a (+) with kanamycin resistance gene, the zone of inhibition is visible only in presence of apramcyin whereas zone of inhibition is absent in both antibiotics in piBR181-Apr, piBRTrc-Apr and piBRUV5-Apr harboring E. coli BL21 (DE3). Figure S5. Cloning strategy for the construction of a recombinant cyanidin-3-O-glucoside (C3G) production cassette in piBRTrc, piBRUV5, and piBR181 multi-monocistronicvector. Two genes anthocyanidin synthase (PhANS) and flavonol-3-O-glycosyltransferase (At3GT) were cloned together to generate the strains S1, S2, S3, S4, S5, and S6. Figure S6. Final assembly of cyanidin-3-O-glucoside system using the previously constructed UDP-d-glucose biosynthesis system in piBR181 to which the anthocyanidin synthase (PhANS) and flavonol-3-O-glycosyltransferase (At3GT) were further [file 12934_2019_1056_MOESM1_ESM.doc]
